# An elderly man having large pulmonary mass and chest pain

**DOI:** 10.4103/1817-1737.62477

**Published:** 2010

**Authors:** Ramakant Dixit, Manoj Arya, A. R. Paramez, Dilip Singh Rathore

**Affiliations:** *Department of Pulmonary Medicine and Tuberculosis, J. L. N. Medical College, Ajmer, India*

A 65-year-old nonsmoker priest presented with a history of dry cough and right-sided chest pain for last three months. The pain was dull aching and relieved by analgesics. He denied any history of fever, expectoration or breathlessness. His medical history was negative.

On physical examination, he was well-built with no clubbing and lymphadenopathy. Respiratory system examination revealed slight fullness on the right side with dull percussion note and decreased pneumophonation in right upper lung fields. The trachea was shifted to the left side. Other body system examination was unrewarding.

His investigation revealed normal blood count and blood biochemistry. Induced sputum was negative for acid-fast bacilli. Chest radiography revealed a large well-defined mass with homogenous consistency occupying almost upper two-thirds of the right lung field with a shift of mediastinum toward the left and no bony erosion [Figure 1]. His electrocardiogram and ultrasound of the abdomen was reported as normal. Computed tomography scan of the thorax could not be done. In view of a possible neoplastic lesion on clinical and radiological assessment, FNAC of the lesion was performed that revealed mostly hemorrhagic aspirate with few atypical cells. For definitive diagnosis, trucut lung biopsy under ultrasound guidance was done, which on histological examination revealed well-defined tubular structures surrounded by undifferentiated mesenchymal tissue consisting of oval- to spindle-shaped cells with hyperchromatic nuclei [Figure 2]. Before a definitive therapy could be planned, the patient had an episode of massive haemoptysis from which he could not be revived.

**Figure 1:**
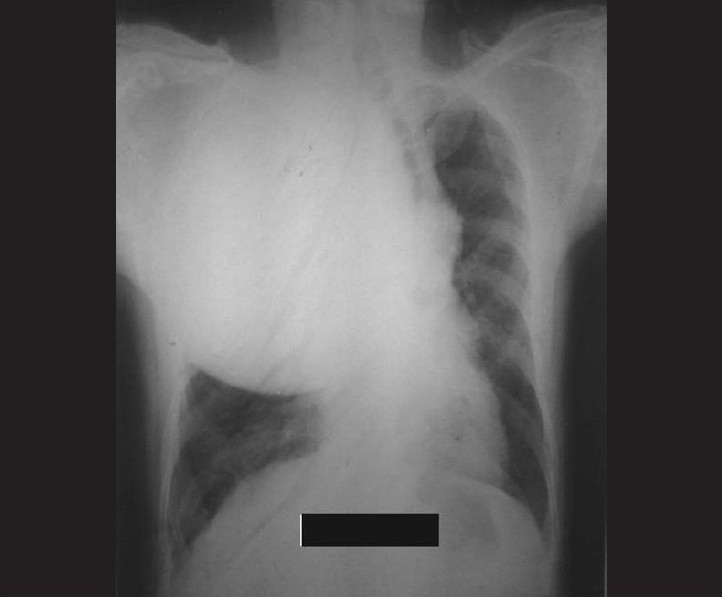
Skiagram chest showing a large mass lesion occupying the right upper and mid zone with mediastinal shift to opposite side

**Figure 2:**
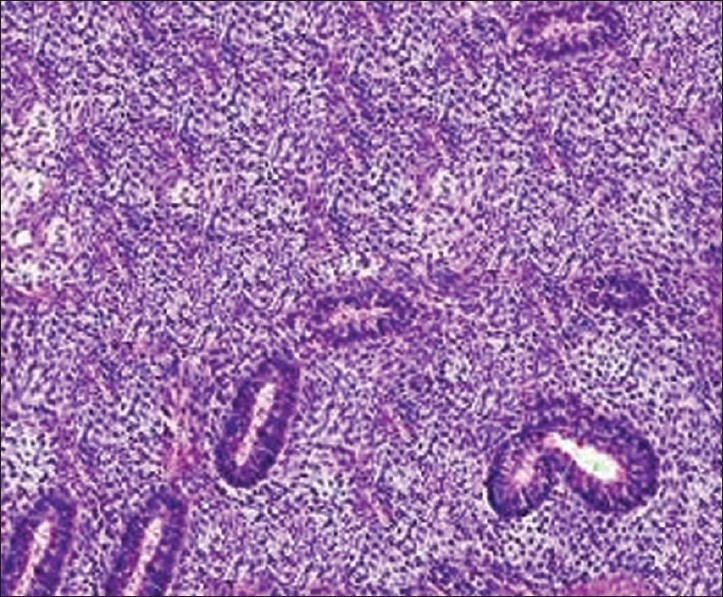
Photomicrograph of tumor biopsy showing oval- to spindle-shaped cells with hyperchromatic nuclei representing mesenchymal stroma and tubular structures representing epithelial component (H and E, ×400).

## QUESTIONS

What is your diagnosis?

Discuss the features and management of this condition?

### Answers

1. Pulmonary blastoma
2. Pulmonary blastoma is a rare tumor that resembles fetal lung of gestational age of 10-12 weeks.[1] It has been described in adults aged 15-77 years with a slight male predominance.[2] Most of these tumors are well circumscribed, intrapulmonary or subpleural and exhibit slow growth. Few cases have been found in association with pulmonary cystic disease.[3]

The clinical presentation is nonspecific and varies from no symptoms to chest symptoms and metastasis symptoms. Chest symptoms include cough, hemoptysis, chest pain, etc. Preoperative diagnosis is usually difficult because chest radiograph, sputum cytology and bronchoscopy are usually unrewarding.[4] It is interesting to note that the first case of pulmonary blastoma reported by Barnard[5] was clinically diagnosed as hydatid cyst and the definitive diagnosis was possible only after surgical excision.

The exact histogenesis of this tumor is controversial. It has been termed as embryoma, blastoma and carcinosarcoma, with the blastoma being the most commonly used term.[5] It is not exactly known whether the tumor arises only from the endoderm or endoderm and mesoderm. It is also not clear whether rather benign looking blastoma and frank carcinosarcoma really belong to one group as two ends of spectrum or represent an entirely different lesion.[4]

Surgical resection is the treatment of choice in most cases. Radiotherapy has been used as a mode of treatment in primary tumor and in metastasis. The role of postoperative adjuvant chemotherapy, irradiation or both is poorly defined and the prognosis is uncertain as the number of cases reported thus far has been small and method of treatment varied.[2] The behavior of the tumor is variable. The longest survival reported after resection is 15 years and the shortest is 3 months. The size of the tumor bears no positive relations to long-term survival.[6]
